# Impact of Skeletal Muscle Depletion on Patients with Myelodysplastic Syndrome Treated with Azacitidine

**DOI:** 10.3390/hematolrep16010012

**Published:** 2024-02-28

**Authors:** Eri Takada, Nobuhiko Nakamura, Yuto Kaneda, Kenji Fukuno, Shin Lee, Kei Fujita, Tetsuji Morishita, Yoshikazu Ikoma, Takuro Matsumoto, Hiroshi Nakamura, Junichi Kitagawa, Nobuhiro Kanemura, Senji Kasahara, Takeshi Hara, Hisashi Tsurumi, Masahito Shimizu

**Affiliations:** 1 Department of Hematology, Gifu-Seino Medical Center, Ibi Kosei Hospital, Gifu 501-0619, Japan; takada.eri.w8@s.gifu-u.ac.jp; 2 Department of Hematology and Infectious Disease, Gifu University Hospital, Gifu 501-1194, Japan; kaneda.yuto.u7@f.gifu-u.ac.jp (Y.K.); yikoma@hematology.ichinaika.gifu.jp (Y.I.); matsumoto.takuro.a1@f.gifu-u.ac.jp (T.M.); nakamura.hiroshi.p5@f.gifu-u.ac.jp (H.N.); kanemura.nobuhiro.t3@f.gifu-u.ac.jp (N.K.); tsurumi.hisashi.v1@f.gifu-u.ac.jp (H.T.); shimizu.masahito.j1@f.gifu-u.ac.jp (M.S.); 3 Department of Hematology, Takayama Red Cross Hospital, Gifu 506-8550, Japan; k-fukuno.wj@takayama.jrc.or.jp; 4 Department of Hematology, Matsunami General Hospital, Gifu 501-6062, Japan; leesin@u-fukui.ac.jp (S.L.); kfujita@u-fukui.ac.jp (K.F.); hara.takeshi.p7@f.gifu-u.ac.jp (T.H.); 5 Department of Hematology, Gifu Municipal Hospital, Gifu 500-8513, Japan; jkita@gmhosp.gifu.gifu.jp (J.K.); skasahara@hematology.ichinaika.gifu.jp (S.K.)

**Keywords:** myelodysplastic syndrome, azacitidine, skeletal muscle depletion, hematological toxicity

## Abstract

Background: Azacitidine (AZA) is the standard treatment for patients with high-risk myelodysplastic syndromes (MDS). The impact of skeletal muscle depletion (SMD), which is associated with outcomes of hematological malignancies, on the clinical course of MDS patients treated with AZA was investigated. Methods: This retrospective, observational study included 50 MDS patients treated with AZA. Muscle mass was evaluated using the skeletal muscle index (SMI), which is the area of muscle mass at the third lumbar vertebra on CT images divided by the square of the height. Results: Of the enrolled patients, 39 were males, and their median age was 69.5 years. Twenty-seven (20 male and 7 female) patients showed SMD. The median survival was 13.4 months in the SMD group and 15.2 months in the non-SMD group, with no significant difference and no significant association between the response rate or severe non-hematological toxicities and the presence of SMD. By contrast, grade 3–4 anemia and thrombocytopenia were significantly more frequent in the SMD group than in the non-SMD group. SMD was associated with severe anemia and thrombocytopenia in MDS patients treated with AZA. Conclusion: Reduced skeletal muscle mass may predict severe hematological toxicity in MDS patients treated with AZA.

## 1. Introduction

Myelodysplastic syndromes (MDS) are a group of neoplastic diseases characterized by abnormal proliferation and apoptosis of hematopoietic cells thought to be caused by abnormalities in immature hematopoietic cells [1]. MDS are more common in older persons, they rarely manifest before age 50 years, and the median age at presentation is 70 years [2]. Therefore, in an aging society, MDS is now a disease that is thought to occur more frequently. The only curative therapy for MDS is allogeneic hematopoietic stem cell transplantation (allo-HSCT), but only a few patients can benefit from this therapy due to the age of the patient population. Thus, the prognosis for MDS patients is generally poor [3].

Azacitidine (AZA), a hypomethylating agent (HMA), prolongs the life expectancy of high-risk MDS patients, but it does not cure them unless allo-HSCT is performed [4]. Therefore, elderly MDS patients for whom allo-HSCT is not indicated must continue AZA administration while maintaining a quality of life without complications. However, older patients are often frail and have many comorbidities that make it difficult to continue the treatment. In fact, almost one-third of high-risk MDS patients treated with HMAs discontinue treatment before four cycles, and nearly half of these patients discontinue after only one cycle [5]. Older age and poor performance status are also reported to be predictors of HMA discontinuation [5]. Continued administration of AZA for MDS requires early identification of the patient group most likely to experience serious adverse events that would cause discontinuation.

The International Prognostic Scoring System (IPSS) and the Revised International Prognostic Scoring System (IPSS-R) are useful prognostic indicators for patients with MDS [6,7]. These systems include disease factors such as bone marrow blasts, chromosomal abnormalities, and blood cell counts, but patient factors must also be considered. Older patients often have poor nutritional status and sarcopenia, which is defined as a loss of skeletal muscle strength and skeletal muscle mass [8]. Malnutrition and sarcopenia have been reported to be associated with prognoses in hematological malignancies, such as acute myeloid leukemia (AML), diffuse large B-cell lymphoma (DLBCL), and multiple myeloma (MM) [9,10]. Decreased skeletal muscle mass itself has also been reported as an independent prognostic factor in male DLBCL patients and adult AML patients [11,12].

Since many patients with MDS are older, they may be more likely to have poor nutritional status and sarcopenia, which could affect treatment outcomes. In fact, nutritional status has been reported to be the prognostic factor for MDS [13]; however, no studies have examined the relevance of sarcopenia or skeletal muscle depletion (SMD) to the prognosis or treatment of MDS. Therefore, the present study focused on the association between MDS and SMD and retrospectively examined whether decreased skeletal muscle mass affects treatment outcomes in MDS patients treated with AZA. In particular, since the development of severe myelosuppression is often a problem in the treatment of MDS with AZA [14,15], the effect of reduced skeletal muscle mass on hematological toxicity was also evaluated.

## 2. Materials and Methods

### 2.1. Patient Cohort and Treatment

This was a retrospective, observational study. Patients aged 18 years or older who started AZA treatment at the Gifu University Hospital and Takayama Red Cross Hospital between March 2011 and November 2020 were included. The definition of MDS was based on the World Health Organization (WHO) classification 2016 [16], and the FAB classification was also evaluated [17]. Baseline variables included age, height, weight, Eastern Cooperative Oncology Group performance status (ECOG PS), peripheral blood cell count, percentage of bone marrow blasts, and red blood cell transfusion dependency. Nutritional status was assessed by the geriatric nutritional risk index (GNRI), calculated from albumin levels and ideal body weight [18]. The Flemish version of the Triage Risk Screening Tool (fTRST) was used for the geriatric assessment [19]. Comorbidities were assessed using the Charlson comorbidity index (CCI) [20]. The prognoses of patients with MDS were classified using IPSS-R [7].

All patients received AZA at 75 mg/m^2^/day, 100 mg/body, or other lower doses for 5 or 7 days on a 28-day cycle. The dosage and number of AZA administration days were determined at each attending physician’s discretion. The Institutional Review Board approved this study, waiving the requirement for written, informed consent because it was a retrospective study (approval 2020-082). This study was conducted according to the human and ethical research principles set forth in the Helsinki guidelines.

### 2.2. Definition of Skeletal Muscle Depletion

**Figure 1:**
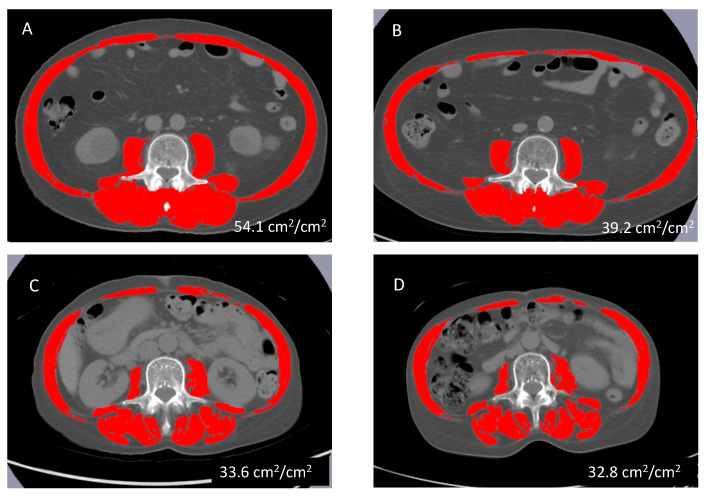
Axial CT image of the third lumbar spine region with skeletal muscles highlighted in red. CT images of a 70-year-old man with non-SMD before (**A**) and after 25 cycles of AZA (**B**); CT images of a 68-year-old woman with SMD before (**C**) and after 11 cycles of AZA (**D**). Abbreviations: CT, computed tomography; SMD, skeletal muscle depletion; AZA, azacitidine.

Pretreatment CT images were analyzed with SliceOMatic software (version 4.3; TomoVision, Montreal, QC, Canada) to calculate the skeletal muscle index (SMI; cm^2^/m^2^). The SMI was calculated by dividing the area of muscle mass at the third lumbar spine on the CT images by the square of the height (Figure 1). The cut-off values for SMD were set for each sex based on a previous report involving Japanese patients (male < 42 cm^2^/m^2^; female < 38 cm^2^/m^2^) [21].

### 2.3. Statistical Analysis

The primary endpoint of this study was overall survival (OS), defined as the time from the start of AZA to death or end of follow-up. Treatment response was assessed according to the revised International Working Group criteria [22]. Response was defined as requiring a duration of 4 weeks. Complete response (CR) was defined as bone marrow blasts less than 5% with normalization of peripheral blood. Bone marrow evaluation was conducted following the normalization of peripheral blood. Partial response (PR) was identified as when peripheral blood normalizes, but bone marrow blasts decrease to below 50% of the baseline level yet remain above 5%. Marrow CR was noted when bone marrow blasts were less than 5% without the normalization of peripheral blood. Stable disease (SD) was defined as not achieving CR, PR, or marrow CR but without evidence of disease progression for at least 8 weeks. Toxicity was also a measured outcome evaluated according to National Cancer Institute-Common Toxicity Criteria, version 2.0.

Continuous variables were expressed as medians and ranges, and group comparisons were made using the Mann–Whitney U test. Categorical variables were expressed as numbers and percentages, and group comparisons were made using the chi-squared or Fisher’s exact test.

The median follow-up was estimated using the reverse Kaplan–Meier method. Survival curves were estimated using the Kaplan–Meier method and compared using the log-rank test. Univariate and multivariable Cox proportional hazards model analyses were performed, including sex, ECOG PS, SMD, and the IPSS-R score as covariates. Multivariable logistic regression analysis was performed to analyze the associations between severe hematological toxicity and AZA dose, days of administration, and SMD. A two-sided *p* < 0.05 was considered significant. Statistical analysis was performed using EZR (version 1.37) [23].

## 3. Results

### 3.1. Patients’ Characteristics and Comparisons between SMD and Non-SMD Group Patients

**Figure 2:**
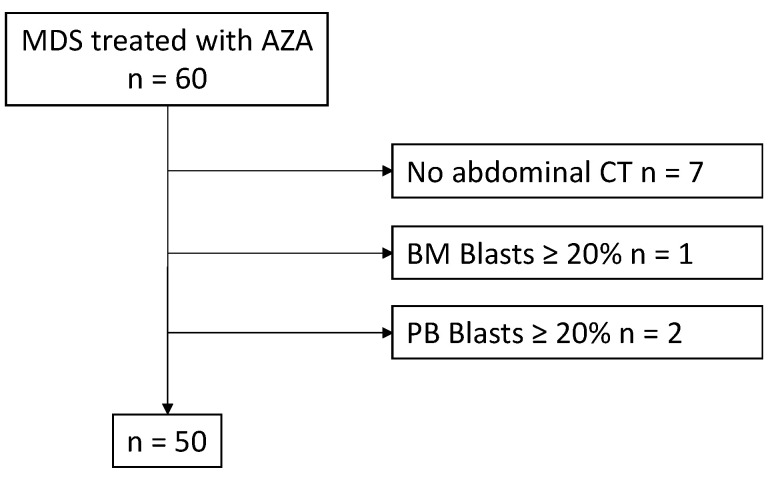
Flow chart of patient selection. Abbreviations: AZA, azacitidine; CT, computed tomography.

A total of 60 patients treated with AZA were enrolled in this study. Of them, ten were excluded (seven who did not undergo CT before AZA treatment, one because of blasts in bone marrow > 20%, and two because of blasts in peripheral blood > 20%), and the remaining fifty patients were included (Figure 2).

The patients’ characteristics are summarized in Table 1. Of the patients enrolled, 11 (22.0%) were female, and the overall median age was 69.5 (17–82) years. The median SMI was 41.9 (range 29.7–59.7) cm^2^/m^2^ for males and 36.8 (range 27.0–43.6) cm^2^/m^2^ for females; it was significantly higher for males (*p* = 0.01). A total of 27 (54.0%) patients were classified into the SMD group, including 20 (51.3%) male and 7 (63.6%) female. Patients in the SMD group tended to be older (*p* = 0.067) and had significantly lower BMI (*p* < 0.001), SMI (*p* < 0.001), and GNRI (*p* = 0.029). By contrast, CCI and fTRST were not significantly different between the two groups. ECOG PS ≥2 was present in 18.5% of patients in the SMD group and in 0% in the non-SMD group (*p* = 0.054). A relationship between increased muscle mass and increased mobility was suggested. However, this association does not suggest a causal relationship, and the direction of this relationship requires further investigation. The SMD group tended to have lower platelet counts (*p* = 0.09) and significantly higher neutrophil counts (*p* = 0.018) than the non-SMD group, but there was no difference in hemoglobin levels. There were no differences between the two groups in FAB classification, WHO classification, and IPSS-R.

The median number of AZA cycles was 5 (range 1–34). The number of patients who completed four or more cycles was 18 (66.7%) in the SMD group and 11 (47.8%) in the non-SMD group, with no significant difference between the two groups. The dose of AZA was 75 mg/m^2^ in 25 patients (50.0%), whereas most of the other patients received 100 mg/body. AZA was administered for 7 days in 15 patients (30.0%) and 5 days in most remaining patients. Seventeen of fifty patients received allo-HSCT, five of twenty-seven (19%) in the SMD group and twelve of twenty-three (52%) in the non-SMD group, with the non-SMD group being significantly more likely to receive allo-HSCT (*p* = 0.01) (Table 1).

### 3.2. Impact of SMD on Overall Survival

**Figure 3:**
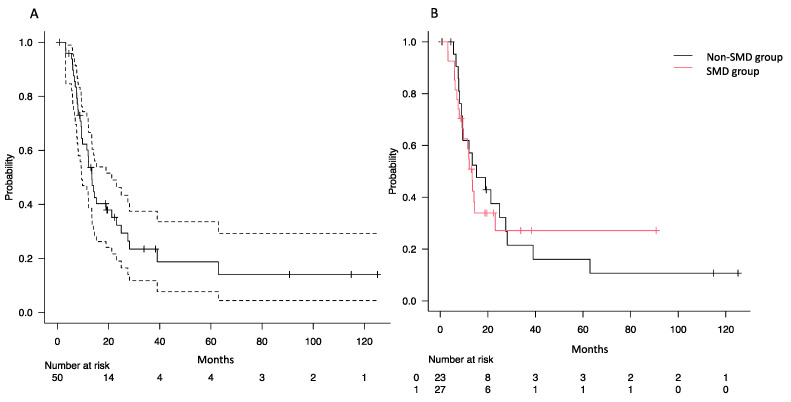
Kaplan–Meier survival curves of overall survival. (**A**) OS in all patients, with the dashed line representing the 95% confidence interval. (**B**) OS with or without SMD. Abbreviations: OS, overall survival; SMD, skeletal muscle depletion.

With a median follow-up time of 38.1 months (interquartile range: 19.6–115.0 months), the median survival of all patients was 13.5 months (Figure 3A). The median survival was 13.4 months in the SMD group and 15.2 months in the non-SMD group, with no significant difference (*p* = 0.897, Figure 3B).

Univariate and multivariable Cox proportional hazards analyzes were performed to identify clinical factors significantly correlated with OS (Table 2). When ECOG PS > 2, SMD, IPSS-R score, and allo-HSCT were included as covariates in these analyses, none of the factors were significantly related to OS on multivariable analysis.

Forty-six patients (92%) discontinued the AZA therapy for various reasons, including disease progression (*n* = 19), toxicity (*n* = 11), death (*n* = 2), and transition to allo-HSCT (*n* = 14). Comparing the reasons for discontinuation of AZA in the SMD and non-SMD groups, in the SMD group, the reasons for discontinuation were disease progression in 14 (52%), adverse events in 7 (26%), death in 2 (7%), and conversion to allo-HSCT in 3 (11%) patients. By contrast, in the non-SMD group, 5 (22%) had disease progression, 4 (17%) had adverse events, and 11 (48%) were referred for allo-HSCT. The data suggest higher incidences of disease progression and death as reasons for the discontinuation of AZA treatment in SMD patients. By contrast, non-SMD patients had a higher rate of conversion to allo-HSCT.

### 3.3. Associations of SMD with Response Rates and Adverse Events

Response rates and serious adverse events are summarized in Table 3. In the total enrolled patients, CR was observed in six (14.6%), PR in three (7.3%), and marrow CR in nine (22.0%) patients. When compared between the SMD and the non-SMD groups, there were no significant differences in the response rate or hematological improvement. On the other hand, the SMD group showed significantly more severe (grade 3 to 4) anemia (48.1% vs. 17.4%, *p* = 0.036) and more severe (grade 3 to 4) thrombocytopenia (63.0% vs. 30.4%, *p* = 0.027).

To identify the factors related to severe hematological toxicity, multivariable logistic regression analysis was performed using SMD plus the dose and duration of AZA administration, which may affect hematological toxicity as covariates. In the SMD group, grade 3–4 anemia [odds ratio (OR) 4.00; 95% CI 1.01–15.80; *p* = 0.048] and thrombocytopenia (OR 4.36, 95% CI 1.17–16.20, *p* = 0.028) were significantly more frequent, but these hematological toxicities were not associated with the dose and duration of AZA administration (Table 4).

Given the observed trend of lower baseline platelet counts in the SMD group than in the non-SMD group, a post hoc analysis was conducted to determine if baseline platelet counts were related to the incidence of severe thrombocytopenia. Multivariate logistic regression analysis, including baseline platelet counts, showed no significant association between pre-treatment platelet counts and severe thrombocytopenia. However, SMD was significantly associated with severe hematological toxicities (Table S1).

## 4. Discussion

AZA is a key agent in high-risk MDS patients who are not candidates for allo-HSCT [14]. Sarcopenia and loss of skeletal muscle have been reported to predict the prognosis for hematological malignancies, such as AML, DLBCL, and MM [10]. In the present study, reduced skeletal muscle mass was not associated with OS or the response rate in MDS patients treated with AZA. By contrast, these patients with loss of skeletal muscle mass showed more severe hematological toxicity, especially anemia and thrombocytopenia.

Severe anemia and thrombocytopenia are significant concerns in patients with MDS. Anemia has been reported to strongly predict cardiovascular outcomes in patients with MDS [24]. A strong relationship has been reported between lower hemoglobin levels and worse cardiovascular outcomes, including cardiac remodeling, congestive heart failure, coronary artery disease, myocardial infarction, arrhythmias, valvular heart disease, and cardiovascular mortality. Similarly, thrombocytopenia in patients with MDS has been reported to be associated with bleeding complications [25]. These complications can range from minor issues, such as subcutaneous bruising and nosebleeds, to more serious conditions, such as gastrointestinal bleeding and even life-threatening intracranial hemorrhage. When administering AZA to MDS patients with reduced skeletal muscle mass, greater attention should be paid to the risk of severe anemia and thrombocytopenia to prevent adverse events and a worse prognosis.

The mechanism by which reduced skeletal muscle mass is associated with severe anemia and thrombocytopenia caused by AZA in MDS patients is unclear, but reduced skeletal muscle mass itself has been reported to affect anemia [26]. It has also been reported that reduced skeletal muscle mass is associated with poorer response to erythropoietin products in hemodialysis patients [27]. These findings may suggest that patients with SMD are more prone to anemia because of their decreased sensitivity to endogenous erythropoietin. The association between skeletal muscle mass and blood cell counts has also been reported in other myeloid malignancies. Improved muscle mass has been shown to be associated with increased hemoglobin levels and less disease progression in patients with myelofibrosis treated with ruxolitinib [28]. These relationships may be affected by a complex interplay of various factors, including energy metabolism, blood metabolism, iron metabolism, and, possibly, unmeasured variables affecting muscle and hematopoietic function. Further research is needed to explore these associations and their implications for treatment response and patient management in MDS.

Previous reports have shown that the prognosis of patients with sarcopenia or reduced skeletal muscle mass volume is poor in DLBCL, AML, and MM [10]. It has been shown that patients with cancers, including breast cancer and gastrointestinal cancer, other than hematological malignancies with skeletal muscle mass depletion also have a poor prognosis [29,30,31]. In addition, in a study evaluating the impact of sarcopenia in 859 allogeneic transplant recipients, including 164 patients with MDS, sarcopenia was identified as an independent predictor of increased risk of non-relapse mortality (HR = 1.58, 95% CI 1.16–2.16, *p* < 0.004) [32]. In the present study, SMD patients had higher incidences of disease progression and death as reasons for discontinuing AZA treatment, but there was no difference in OS or treatment response between the SMD and non-SMD groups. These results may be related to the fact that the present study included MDS patients treated with AZA and the small number of patients (34.0%) who received allo-HSCT. The impacts of skeletal muscle mass on prognosis and response rates in patients with MDS need to be analyzed in a larger number of patients.

Direct intervention measures for sarcopenia are limited, but rehabilitation and nutritional supplements may improve sarcopenia [33]. Rehabilitation and nutritional supplements are also useful in improving prognosis and preventing adverse events in patients with hematological malignancies undergoing chemotherapy [34,35]. Furthermore, it has also been reported that poorer OS is strongly related to new-onset sarcopenia following radiotherapy in hepatocellular carcinoma patients, and they require strong nutritional support throughout radiotherapy to prevent the onset of sarcopenia after therapy [30]. In practice, it is difficult to provide exercise interventions that increase skeletal muscle mass in patients with malignant disease, and few reports have shown that an increase in muscle mass itself leads directly to improved prognosis. However, a randomized, controlled trial of 54 patients with incurable cancer showed that exercise therapy and nutritional rehabilitation interventions are useful, even if they do not provide significant improvements in muscle mass or physical activity [36]. There are also reports suggesting that AZA may have an effect on muscle regeneration at the cellular level [37]. On the other hand, muscle pain, cramps, and weakness have been listed as side effects of AZA treatment [38]. This suggests that AZA treatment may have some negative effects on muscles, and exercise therapy and nutritional rehabilitation interventions for skeletal muscle depletion are also crucial during AZA treatment. It is necessary to develop an effective and safe exercise therapy for MDS patients with sarcopenia because no clinical trials have been conducted on these patients.

There are several limitations to this study. First, this study was retrospective and may have had selection bias. Second, the number of patients included in this study was not sufficient, and this study has several statistical power constraints. Therefore, although there was no statistically significant effect of SMD on prognosis in the present study, we believe that further studies with larger sample sizes are needed to obtain more definitive findings. A notable limitation was the lack of longitudinal data on skeletal muscle mass. The study design did not allow for the assessment of changes in muscle mass over time, which could have a significant impact on treatment outcomes. These limitations are mainly because not all MDS patients treated with AZA necessarily underwent CT scans. Another limitation was the present study’s lack of focus on how age-related impairments might affect AZA treatment outcomes in MDS patients. The absence of a detailed analysis of factors, such as declining organ function and polypharmacy due to aging, may limit the applicability of the present findings to older MDS populations. In addition, heterogeneity in IPSS scoring, duration of AZA treatment, and the use of allo-HSCT are also limitations. Thus, cautious interpretation of the present results is required. Further studies with a larger number of patients may be needed.

## 5. Conclusions

In conclusion, reduced skeletal muscle mass is not a prognostic factor, but it may predict severe hematological toxicity in MDS patients treated with AZA. In particular, severe anemia and thrombocytopenia should be noted when administering AZA to high-risk MDS patients with SMD. Since severe hematological toxicity in MDS patients treated with AZA leads to treatment discontinuation and poor prognosis, predicting these adverse events in advance may be necessary when managing therapy. For instance, early intervention for iron overload may be considered in these patients because they may require frequent blood transfusions. Consideration of AZA dose reduction may also help avoid hematological toxicity. Assessments of sarcopenia and skeletal muscle mass may lead to treatment options that do not compromise patients’ quality of life.

## Figures and Tables

**Table 1:** Patients’ characteristics and comparisons between the SMD and non-SMD group patients.

Factor	Group	Overall	SMD	Non-SMD	*p*-Value
		(*n* = 50)	(*n* = 27)	(*n* = 23)	
Female, *n* (%)		11 (22.0)	7 (25.9)	4 (17.4)	0.515
Age at AZA start, y, median (range)		69.5 (17–82)	71.0 (28.0–82.0)	67.00 (17.0–79.0)	0.067
Duration between diagnosis to AZA start, days, median (range)		32.5 (0-1405)	31 (0–781)	36 (4–1405)	0.495
BMI, kg/m^2^, median (range)		22.65 (16.0–28.9)	21.0 (16.0–27.1)	24.4 (19.3–28.9)	<0.001
SMI, cm^2^/m^2^, median (range)		40.0 (27.0–59.7)	37.38 (27.0–41.9)	46.8 (38.2–59.7)	<0.001
GNRI, median (range)		102.2 (67.0–123.8)	99.5 (67.0–123.8)	106.1 (83.5–120.5)	0.029
CCI, *n* (%)	0–1	30 (60.0)	15 (55.6)	15 (65.2)	0.569
	≥2	20 (40.0)	12 (44.4)	8 (34.8)	
fTRST, *n* (%)	0	22 (44.0)	10 (37.0)	12 (52.2)	0.199
	1	22 (44.0)	15 (55.6)	7 (30.4)	
	2	6 (12.0)	2 (7.4)	4 (17.4)	
ECOG PS, *n* (%)	0–1	45 (90.0)	22 (81.5)	23 (100.0)	0.054
	≥2	5 (10.0)	5 (18.5)	0 (0.0)	
Neutrophil count, ×10^3^/μL, median (range)		1.1 (0.89–388.64)	1.58 (0.52–388.64)	0.92 (0.89–19.58)	0.018
Hemoglobin, g/dL, median (range)		8.2 (4.7–12.40)	8.1 (4.7–12.1)	8.3 (5.0–12.4)	0.884
Platelet count, ×10^3^/μL, median (range)		66.5 (7.0–579.0)	54.0 (7.0–280.0)	101.0 (17.0–579.0)	0.090
BM percentage blasts, median (range)		9.45 (1.7–18.3)	9.4 (1.9–18.3)	9.5(1.7–17.9)	0.846
Red cell transfusion dependence, *n* (%)		11 (22.0)	5 (18.5)	6 (26.1)	0.733
FAB classification, *n* (%)	RA	5 (10.0)	3 (11.1)	2 (8.7)	1.000
	RAEB	45 (90.0)	24 (88.9)	21 (91.3)	
WHO classification, *n* (%)	SLD	2 (4.0)	0 (0.0)	2 (8.7)	0.402
	MLD	1 (2.0)	1 (3.7)	0 (0.0)	
	RS-MLD	1 (2.0)	1 (3.7)	0 (0.0)	
	MDS-U	1 (2.0)	1 (3.7)	0 (0.0)	
	EB-1	19 (38.0)	9 (33.3)	10 (43.5)	
	EB-2	26 (52.0)	15 (55.6)	11 (47.8)	
IPSS-R, *n* (%)	Low	1 (2.0)	0 (0.0)	1 (4.3)	0.518
	Intermediate	5 (10.0)	4 (14.8)	1 (4.3)	
	High	17 (34.0)	9 (33.3)	8 (34.8)	
	Very high	27 (54.0)	14 (51.9)	13 (56.5)	
AZA ≥ 4 cycles, *n* (%)		29 (58.0)	18 (66.7)	11 (47.8)	0.252
Dose of AZA, *n* (%)	75 mg/m^2^	25 (50.0)	10 (37.0)	15 (65.2)	0.167
	100 mg/body	16 (32.0)	11 (40.7)	5 (21.7)	
	other	9 (18.0)	6 (22.2)	3 (13.0)	
Days of AZA treatment, *n* (%)	7 days	15 (30.0)	7 (25.9)	8 (34.8)	
	5–7 days	35 (70.0)	20 (74.1)	15 (65.2)	
Allo-HSCT, *n* (%)		17 (34.0)	5 (18.5)	12 (52.1)	0.012

SMD, skeletal muscle depletion; AZA, azacitidine; BMI, body mass index; SMI, skeletal muscle index; GNRI, geriatric nutritional index; CCI, Charlson comorbidities index; fTRST, Flemish version of the Triage Risk Screening Tool; ECOG PS, Eastern Cooperative Oncology Group performance status; BM, bone marrow; FAB, French–American–British; WHO, World Health Organization; IPSS, International Prognostic Scoring System; IPSS-R, Revised International Prognostic Scoring System; RA, refractory anemia; RAEB, refractory anemia with excess blasts; RAEB-t, refractory anemia with excess blasts in transformation; SLD; single lineage dysplasia; MLD, multilineage dysplasia; RS-MLD, ring sideroblasts with multilineage dysplasia; MDS-U, myelodysplastic syndrome, unclassifiable; EB, excess blast; AML, acute myeloid leukemia; allo-HSCT, allogeneic hematopoietic stem cell transplantation.

**Table 2:** Univariate and multivariable COX proportional hazards analyses of clinical factors significantly associated with overall survival.

	Univariate Analysis		Multivariable Analysis	
Factor	HR (95% CI)	*p*-Value	HR (95% CI)	*p*-Value
ECOG PS ≥ 2	2.73 (0.91–8.16)	0.073	3.15 (0.98–10.17)	0.055
SMD	1.05 (0.54–2.03)	0.897	0.75 (0.36–1.56)	0.456
IPSS-R score	1.19 (0.96–1.48)	0.116	1.21 (0.96–1.53)	0.098
Allo-HSCT	0.59 (0.28–1.25)	0.167	0.56 (0.26–1.26)	0.162

ECOG PS, Eastern Cooperative Oncology Group performance status; IPSS-R, Revised International Prognostic Scoring System; HR, hazard ratio; CI, confidence interval; SMD, skeletal muscle depletion; allo-HSCT, allogeneic hematopoietic stem cell transplantation.

**Table 3:** Response rate and severe adverse events.

Factor	Group	All Patients	SMD	Non SMD	*p*-Value
		(*n* = 50)	(*n* = 27)	(*n* = 23)	
Response, *n* (%)	CR	6 (14.6)	4 (19.0)	2 (10.0)	0.714
	PR	3 (7.3)	2 (9.5)	1 (5.0)	
	Marrow CR	9 (22.0)	4 (19.0)	5 (25.0)	
	SD	15 (36.6)	6 (28.6)	9 (45.0)	
	PD	8 (19.5)	5 (23.8)	3 (15.0)	
Hematological improvement, *n* (%)	HI-E	18 (40.9)	9 (37.5)	9 (45.0)	0.760
	HI-P	16 (50.0)	12 (57.1)	4 (36.4)	0.458
	HI-N	10 (50.0)	3 (42.9)	7 (53.8)	1.000
Severe hematological adverse event, *n* (%)	Neutropenia	29 (58.0)	17 (63.0)	12 (52.2)	0.567
	Thrombocytopenia	24 (48.0)	17 (63.0)	7 (30.4)	0.027
	Anemia	17 (34.0)	13 (48.1)	4 (17.4)	0.036
	FN	21 (42.0)	13 (48.1)	8 (34.8)	0.398
Severe non-hematological adverse event, *n* (%)	Infection	16 (32.0)	10 (37.0)	6 (26.1)	0.546
	Other	6 (12.0)	5 (18.5)	1 (4.3)	0.199

SMD, skeletal muscle depletion; CR, complete remission; PR, partial remission; SD, stable disease; PD, progressive disease; HI, hematological improvement; E, erythrocyte; P, platelet; N, neutrophil; FN, febrile neutropenia; SMD, skeletal muscle depletion.

**Table 4:** Multivariable logistic regression analysis of severe hematological adverse events.

	Neutropenia		Thrombocytopenia		Anemia	
Factor	OR (95% CI)	*p*-Value	OR (95% CI)	*p*-Value	OR (95% CI)	*p*-Value
Dose of AZA: 75 mg/m^2^	0.91 (0.28–3.01)	0.882	1.48 (0.40–5.43)	0.557	0.77 (0.21–2.85)	0.698
Days of AZA treatment: 7 days	0.53 (0.15–1.83)	0.317	0.28 (0.07–1.14)	0.076	0.39 (0.09–1.74)	0.216
SMD	1.45 (0.44–4.78)	0.544	4.36 (1.17–16.20)	0.028	4.00 (1.01–15.80)	0.048

AZA, azacitidine; SMD, skeletal muscle depletion; OR, odds ratio; CI, confidence interval.

## Data Availability

The datasets generated during and analyzed during the current study are available from the corresponding author upon reasonable request.

## Supplementary Materials

The following supporting information can be downloaded at: https://www.mdpi.com/article/10.3390/hematolrep16010012/s1, Table S1: Multivariable logistic regression analysis of severe thrombocytopenia with addition of platelet counts before azacitidine treatment.
