# Supplementary material for: Impact of Skeletal Muscle Depletion on Patients with Myelodysplastic Syndrome Treated with Azacitidine
Source: Hematol Rep. 2024 Feb 28;16(1):114–24. doi: 10.3390/hematolrep16010012 (PMC10970390; doi:10.3390/hematolrep16010012)
Supplement: Supplementary file 1 [file hematolrep-16-00012-s001.zip › hematolrep-2779303-supplementary.pdf]

hematology reports

Supplementary Materials

Table S1. Multivariable logistic regression analysis of severe thrombocytopenia with addition of platelet counts before AZA treatment.

Factor	Thrombocytopenia	
	**OR (95% CI)**	***p* Value**
Dose of AZA: 75 mg/m^2	0.47 (0.12–1.87)	0.282
Days of AZA treatment: 7 days	1.46 (0.40–5.39)	0.570
SMD	4.27 (1.13–16.10)	0.032
Platelet counts < 75 × 10^3/μL	1.12 (0.31–4.07)	0.858

AZA, azacitidine; SMD: skeletal muscle depletion; OR, odds ratio; CI, confidence interval.
